# *Bacillus pumilus* TS1 alleviates *Salmonella* Enteritidis-induced intestinal injury in broilers

**DOI:** 10.1186/s12917-023-03598-0

**Published:** 2023-02-10

**Authors:** Yinkun Liu, Zixin Li, Hao Li, Shuangshuang Wan, Shu Tang

**Affiliations:** College of Veterinary Medicine, Nanjing Agricultural University, Nanjing, 210095 China

**Keywords:** *Bacillus pumilus*, HIF-1α, Histopathology, HSP70, Inflammatory cytokines, Oxidative stress, PKC, MAPK, Probiotics

## Abstract

**Background:**

In the current context of reduced and limited antibiotic use, several pathogens and stressors cause intestinal oxidative stress in poultry, which leads to a reduced feed intake, slow or stagnant growth and development, and even death, resulting in huge economic losses to the poultry breeding industry. Oxidative stress in animals is a non-specific injury for which no targeted drug therapy is available; however, the health of poultry can be improved by adding appropriate feed additives. *Bacillus pumilus*, as a feed additive, promotes growth and development and reduces intestinal oxidative stress damage in poultry. Heat shock protein 70 (HSP70) senses oxidative damage and repairs unfolded and misfolded proteins; its protective effect has been widely investigated. Mitogen-activated protein kinase/protein kinase C (MAPK/PKC) and hypoxia inducible factor-1 alpha (HIF-1α) are also common proteins associated with inflammatory response induced by several stressors, but there is limited research on these proteins in the context of poultry intestinal *Salmonella* Enteritidis (SE) infections. In the present study, we isolated a novel strain of *Bacillus pumilus* with excellent performance from the feces of healthy yaks, named TS1. To investigate the effect of TS1 on SE-induced enteritis in broilers, 120 6-day-old white-feathered broilers were randomly divided into four groups (con, TS1, SE, TS1 + SE). TS1 and TS1 + SE group chickens were fed with 1.4 × 10^7^ colony-forming units per mL of TS1 for 15 days and intraperitoneally injected with SE to establish the oxidative stress model. Then, we investigated whether TS1 protects the intestine of SE-treated broiler chickens using inflammatory cytokine gene expression analysis, stress protein quantification, antioxidant quantification, and histopathological analysis.

**Results:**

The TS1 + SE group showed lower MDA and higher GSH-Px, SOD, and T-AOC than the SE group. TS1 alleviated the effects of SE on intestinal villus length and crypt depth. Our results suggest that SE exposure increased the expression of inflammatory factors (IL-1β, IL-6, TNF-α, IL-4, and MCP-1), p38 MAPK, and PKCβ and decreased the expression of HSP60, HSP70, and HIF-1α, whereas TS1 alleviated these effects.

**Conclusions:**

*Bacillus pumilus* TS1 alleviated oxidative stress damage caused by SE and attenuated the inflammatory response in broilers through MAPK/PKC regulation of HSPs/HIF-1α.

**Supplementary Information:**

The online version contains supplementary material available at 10.1186/s12917-023-03598-0.

## Background

In the current context of reducing and limiting antibiotic use [1], several pathogens and stressors cause intestinal oxidative stress in poultry, especially in the first month of life when the immune system is underdeveloped, which leads to major economic losses [2]. In the poultry industry, several factors such as pathogens, environmental factors, and breeding density affect poultry health, which easily leads to oxidative stress and inflammation in the intestinal tract of poultry. Probiotics have been widely used in the prevention of human or animal intestinal inflammatory diseases [3]. In the poultry industry, probiotics are used as feed additives and have been demonstrated to improve the growth performance of broilers [4, 5], improve the feed conversion ratio, positively affect the activity of the intestinal microbiota [6], and reduce *Salmonella* Enteritidis (SE) in newborn chicks [7]. By occupying the intestinal niche as the dominant flora, probiotic bacteria alleviate the effect of pathogenic bacteria on poultry [8] and promote weight gain in broilers [9] and thus can be used as an alternative to antibiotics [10]. According to previous reports, *Bacillus* spp. exhibit high acid and bile salt tolerance and high temperature resistance and survive and grow in the intestine to form biofilms and secrete antimicrobials [11]. Although several potentially probiotic *Bacillus* strains have been reported, the number of *Bacillus* strains used in animal feed, especially in poultry feed, is limited [12]. *Bacillus pumilus*, which is approved for use as a feed additive in poultry diets in China, improves the composition of the cecal microflora of poultry birds at an early age, stimulates the immune system, and produces antimicrobial compounds to protect the body against pathogen attack [13–18]; therefore, the present study aimed to investigate whether our TS1 alleviates SE-induced oxidative and inflammatory damage in poultry.Oxidative stress occurs when the body is subjected to various harmful stimuli [19–21], which lead to elevated levels of highly active molecules in the body, such as reactive oxygen species (ROS)[22] and reactive nitrogen [23]. Under these conditions, the oxide-scavenging capacity of cells is significantly lower than the antioxidant capacity necessary against the existing degree of oxidation [24]. The imbalance between the oxidation system and antioxidant system leads to the damage of cells and important biomolecules [25]. Oxidative stress has a particularly significant effect on poultry intestines [26]; specifically, it decreases the activity of intestinal superoxide dismutase (SOD) [27], glutathione peroxidase (GSH-Px) [28], and total antioxidant capacity (T-AOC) and increases malondialdehyde (MDA) content [29]. Moreover, the molecular structure of sugars, lipids, and proteins undergo structural changes [24], leading to oxidative damage and increasing the permeability of the intestinal mucosal epithelium [30]. The increased expression of intestinal inflammatory factors such as interleukin-1β (IL-1β) [31], IL-4, IL-6 [29], and tumor necrosis factor (TNF-α) further induces more severe mucosal injury [32]. Finally, these effects lead to a reduced feed intake, slow or stagnant growth and development, and even death, resulting in huge economic losses to the poultry breeding industry.

Under oxidative stress, a protective mechanism called heat shock response (HSR) is induced [33], which typically comprises changes in the expression of heat shock protein 70 (HSP70), a key member of the highly conserved heat shock protein family [34, 35]. HSP70 senses oxidative damage and repairs unfolded and misfolded proteins [36]; its protective effect on the body has been widely investigated [37]. It was reported that dietary supplementation of probiotics in poultry also causes changes in HSP70 and has a protective effect on the body [28]. HSP70 has also been considered as a therapeutic target [38]. Under oxidative stress, HSP70 outside and inside intestinal tissue cells shows differential expression; HSP70 expression decreases within cells but increases in the extracellular environment [39].

The mitogen-activated protein kinase/protein kinase C (MAPK/PKC) pathway is a common inflammatory response pathway induced by several stresses [29]. However, research on the MAPK/PKC pathway in poultry remains limited. Hypoxia-inducible factor-1 alpha (HIF-1α) is widely involved in hypoxia-induced specific responses in cells and is closely associated with cell damage; it plays an important role in the regulation of energy under insufficient oxygen supply [40]. Therefore, in the present study, we aimed to investigate the expression of HSP, MAPK/PKC, and HIF-1α proteins and determine whether TS1 mitigates SE-induced oxidative stress injury. In this study, broilers were first fed with TS1 for 15 days. SE infection is a common phenomenon in poultry breeding, and bacterial infections also induce stress, which reduces the height of intestinal villi and number of goblet cells, promotes intestinal cell apoptosis, induces intestinal mucosal damage, and endangers poultry health [41]. SE-induced overproduction of ROS and oxidants may cause epithelial cell damage and other histopathological changes; therefore, this study established an oxidative stress model by intraperitoneal injection of SE. We hypothesized that TS1 attenuates SE-induced intestinal oxidative stress damage in broiler chickens by altering the protein expression levels of MAPK/PKC, HSP70, and HIF-1α.

## Results

### TS1 alleviated *Salmonella* Enteritidis (SE)-induced inflammation in intestinal tissue

**Fig. 1:**
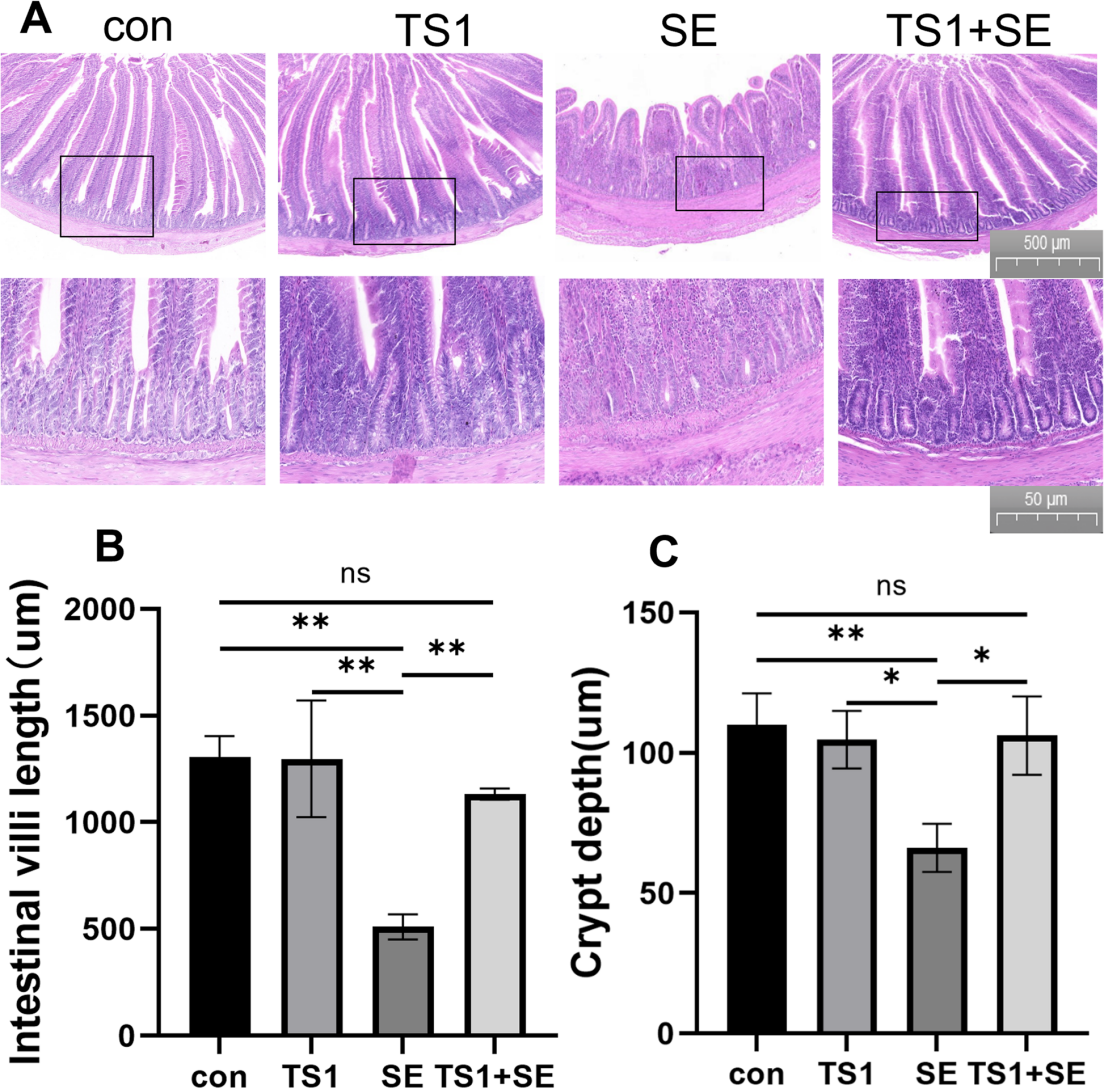
TS1 ameliorated the shortening of the intestinal villi and crypt depth caused by *Salmonella* Enteritidis (SE). **A** Hematoxylin/eosin staining of intestinal tissue (**B**) Histological analysis of small intestinal villus length. **C** Histological analysis of small intestinal crypt depth. All values are expressed as mean ± standard deviation. Asterisks indicate significant differences (^ns^*P* > 0.05, *P < 0.05, and **P < 0.01) between the control (con) and treatment groups

To demonstrate whether TS1 alleviates SE-induced intestinal histological changes, intestinal tissue sections were observed. Pathological changes in the intestinal structure were observed following inflammation (Fig. 1). Jejunal tissue morphology in the control (con) group and TS1 group appeared normal. However, the jejunal tissue of chickens in the SE group showed histopathological changes. Severe cell damage was visible under the microscope, including marked shortening of the intestinal villi, reduction in crypt depth, and thickening of the submucosa. In the TS1 + SE group, the villus length and crypt depth were significantly higher than those in the SE group, and the submucosal thickening was significantly improved. No significant differences were observed between the con group and TS1 group.

**Fig. 2:**
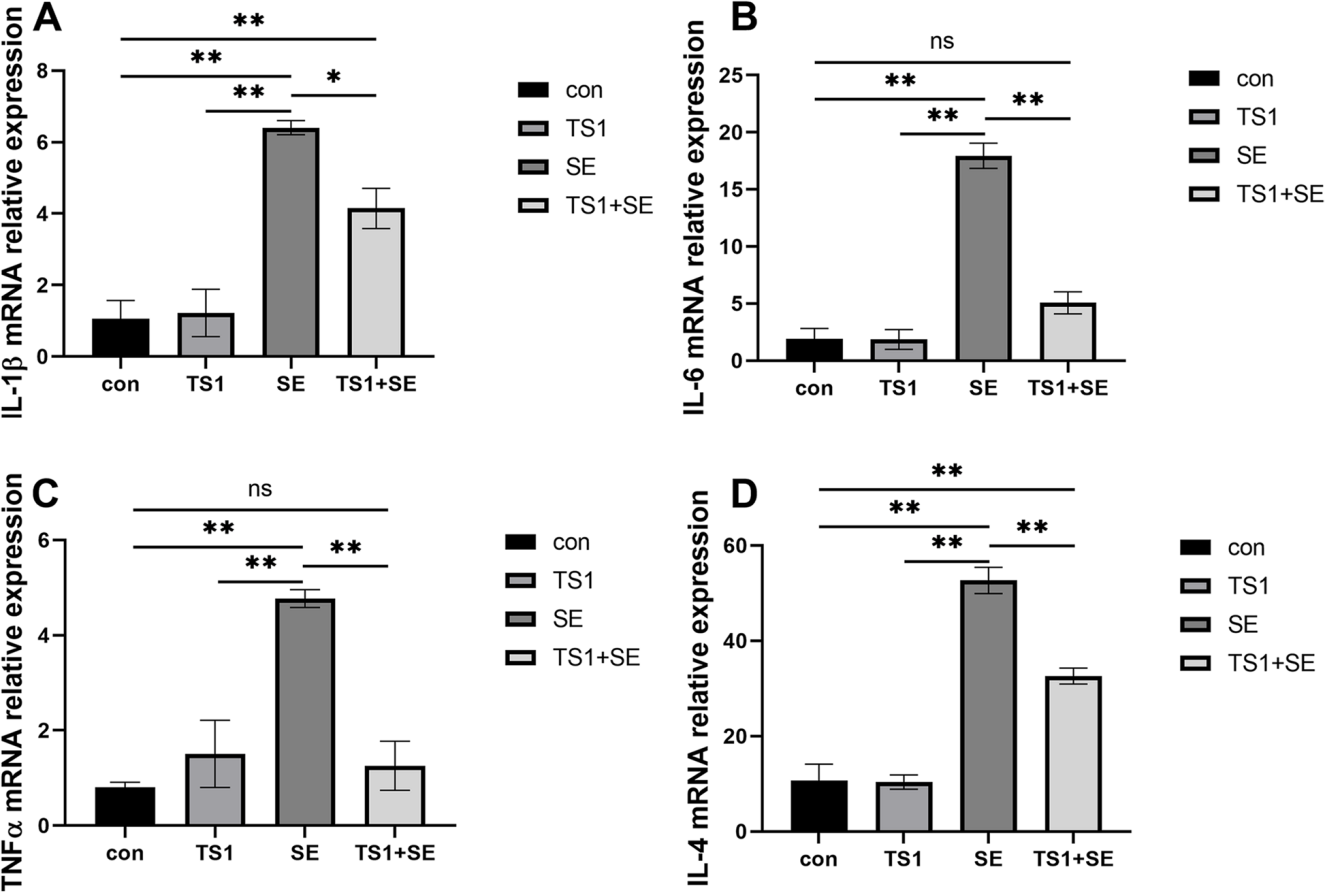
Effects of *Salmonella* Enteritidis (SE) and TS1 on the mRNA level of inflammation-related genes in chicken intestine. The mRNA expression of interleukin (*IL*)-1β (**A**), *IL-6* (**B**), tumor necrosis factor (*TNF*)-α (**C**), and *IL-4* (**D**). All values are expressed as mean ± standard deviation. Asterisks indicate significant differences (*P < 0.05 and **P < 0.01) between the control (con) and treatment groups

Potent inflammatory mediators, such as IL-1β, IL-6, TNFα, and IL-4, were detected by reverse transcription quantitative PCR (RT-qPCR) in jejunal tissue (Fig. 2A-D). The primer sequences used for RT-qPCR are listed in Table 1. The expression levels of inflammatory mediators in the SE group were the highest among the four groups, and TS1 supplementation significantly reduced the SE-induced abnormal increase in the expression levels of inflammatory mediators (*P* < 0.05). There were significant differences in the expression levels of inflammatory mediators, except TNFα, between the TS1 + SE and con groups. There were no significant differences observed between the con and TS1 groups. Together, these results suggest that TS1 significantly alleviates SE-induced inflammation in intestinal tissue.

**Table 1:** Genes and primers used in this study

Target gene	Primer sequence(5’ – 3’)	Product size
IL-1β	Forward:GTACCGAGTACAACCCCTGC Reverse:AGCAACGGGACGGTAATGAA	98
IL-6	Forward:CCTCCTCGCCAATCTGAAGTCA Reverse: GGACAGCCTATGCCAACAAG	210
TNFα	Forward:ACACGACAGCCAAGTCAACG Reverse:ACACGACAGCCAAGTCAACG	168
IL-4	Forward:GTGCCCACGCTGTGCTTAC Reverse:AGGAAACCTCTCCCTGGATGTC	82
HSP70	Forward:CGGGCAAGTTTGACCTAA Reverse:TTGGCTCCCACCCTATCTCT	120
β-actin	Forward:TTGGTTTGTCAAGCAAGCGG Reverse:CCCCCACATACTGGCACTTT	100

### TS1 reduced the expression of inflammatory mediators in chicken serum

**Fig. 3:**
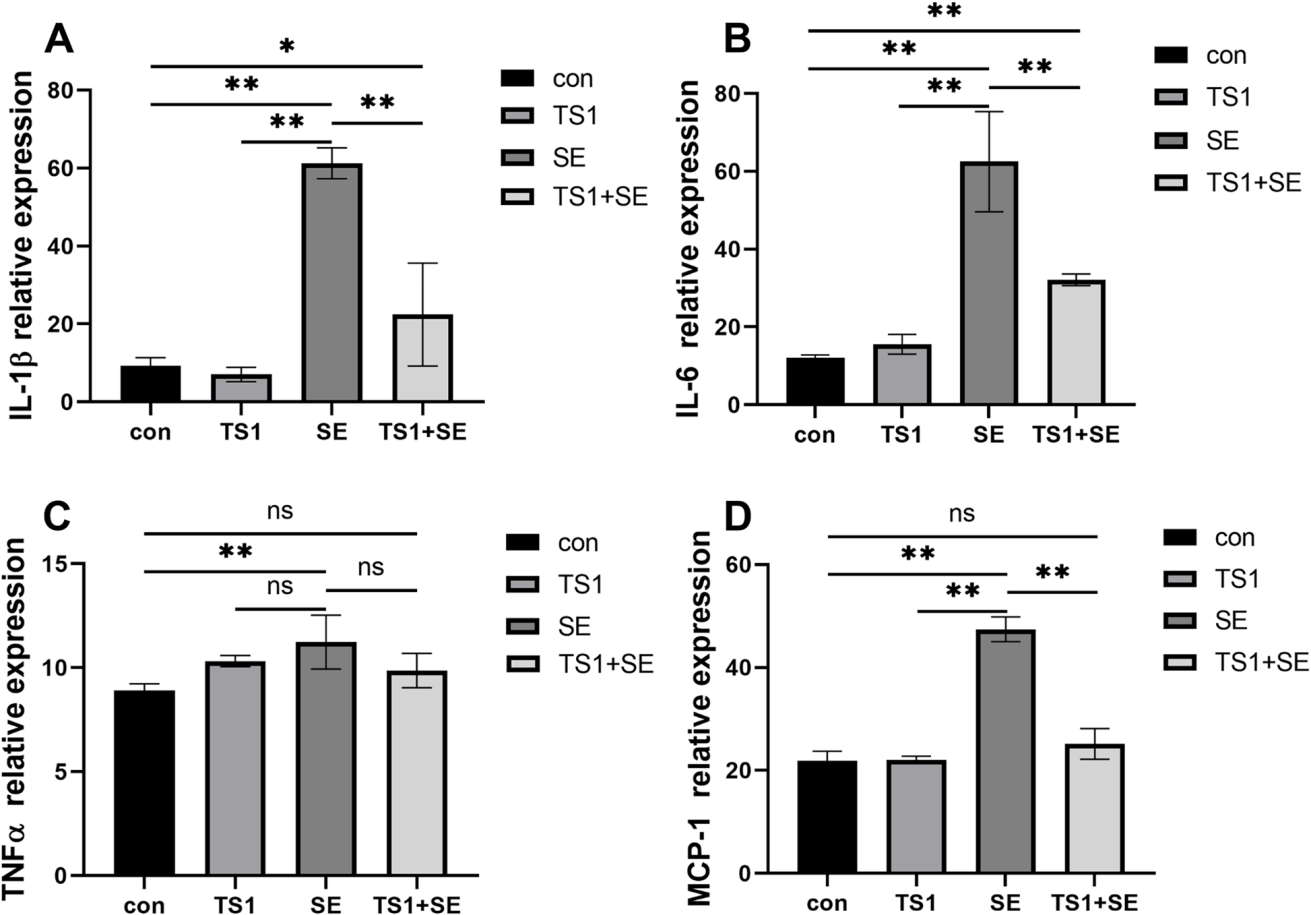
Effects of *Salmonella* Enteritidis (SE) and TS1 on the level of inflammation-related proteins in chicken serum. Relative protein expression of interleukin (IL)-1β (**A**), IL-6 (**B**), tumor necrosis factor (TNF)-α (**C**), and monocyte chemoattractant protein (MCP)-1 (**D**). All values are expressed as mean ± standard deviation. Asterisks indicate significant differences (^ns^*P* > 0.05, *P < 0.05, and **P < 0.01) between the control (con) and treatment groups

The SE group showed significantly higher serum IL-1β (Fig. 3A), IL-6 (Fig. 3B), TNFα (Fig. 3C), and MCP-1 (Fig. 3D) than the con group, whereas the TS1 + SE group showed significantly lower serum levels of proteins than the SE group. However, TNF-α levels (Fig. 3C) in the TS1 + SE group were lower than those in the SE group (*P* > 0.05). There were no significant differences between the TS1 group and con group (*P* > 0.05). Taken together, these results suggest that 1.4 × 10^7^ CFU/mL of TS1 protects broiler chickens from the inflammation induced by SE.

### TS1 alleviated *Salmonella* Enteritidis (SE)-induced oxidative stress in chicken serum and clinical signs in broilers

**Fig. 4:**
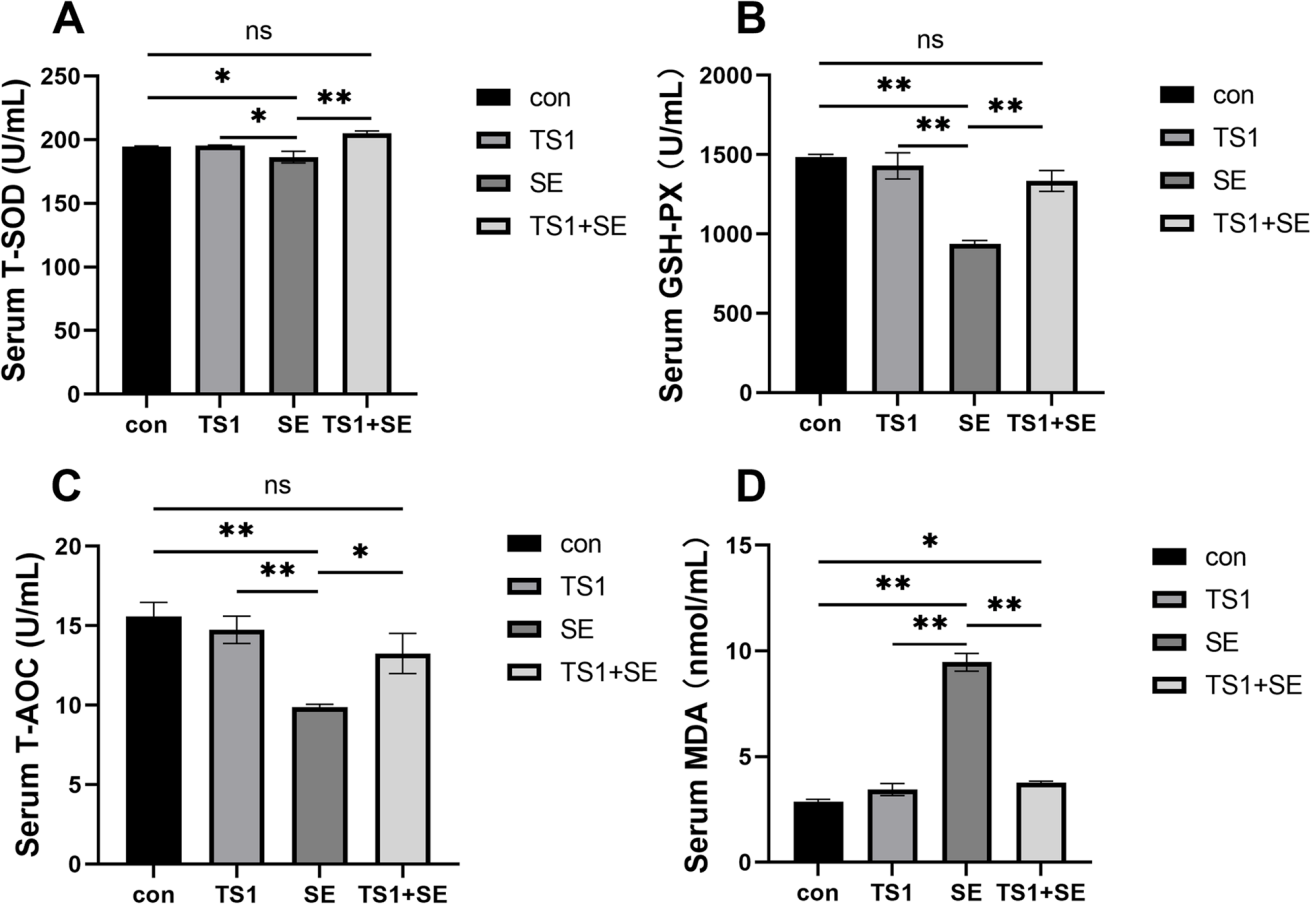
Effects of *Salmonella* Enteritidis (SE) and TS1 on oxidative stress indexes in chicken serum. The relative levels of total superoxide dismutase (T-SOD; **A**), glutathione peroxidase (GSH-Px; **B**), total antioxidant capacity (T-AOC; **C**), and malondialdehyde (MDA; **D**). All values are expressed as mean ± standard deviation. Asterisks indicate significant differences (*P < 0.05 and **P < 0.01) between the control (con) and treatment groups

The activity of the antioxidant enzymes SOD and GSH-Px (Fig. 4A-B) and the T-AOC (Fig. 4C; *P* < 0.05) was significantly lower in the SE group than in the con group. In the TS1 + SE group, the T-AOC and activity of SOD and GSH-Px were higher (*P* < 0.05) than those in the SE group. Additionally, MDA content (Fig. 4D) in the SE group was significantly higher (*P* < 0.05) than that in the other groups. There was also no significant difference between the con group and the TS1 and TS1 + SE groups (*P* > 0.05). In general, these findings indicate that TS1 alleviated oxidative stress caused by SE.

The main symptoms shown by broiler chickens in the SE group were drooping wings, disordered feathers, decreased feed intake, depression, severe diarrhea, and green feces after SE injection. The hock and hip joints of individual diseased chickens were swollen to different degrees, which affected the normal walking of the chickens, and the diseased chickens lay down for long periods. Broilers in the TS1 + SE group after SE injection also showed drooping wings, disordered feathers, decreased feed intake, and depression, but the diarrhea was less severe than that observed in the SE group, with diseased chickens showing green feces and no joint swelling. In contrast, broilers in the con and TS1 groups injected with phosphate-buffered saline (PBS) did not show the above symptoms, and their behavior remained the same as before the injection.

### TS1 improved the expression of HSP70 in intestinal tissue of chicken treated with *Salmonella* Enteritidis (SE)

**Fig. 5:**
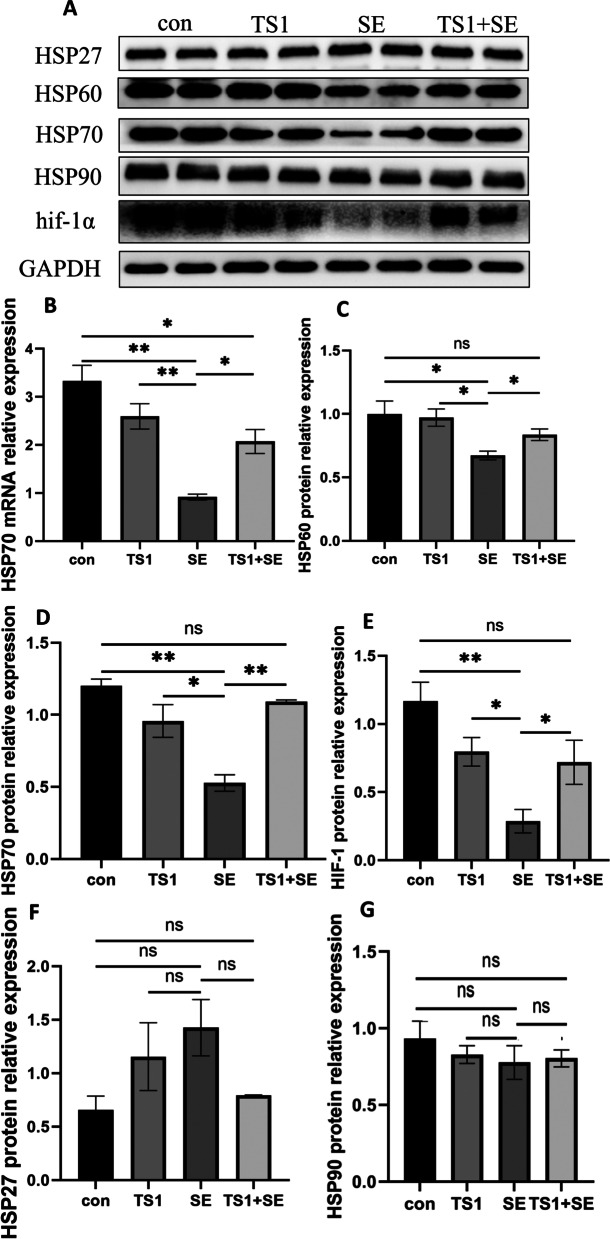
TS1 improved the low expression of HSPs and HIF-1α. Relative mRNA expression of HSP70 (**B**) and the relative protein expression of HSP60 (**A**, **C**), HSP70 (**A**, **D**), hif-1α (**A**, **E**), HSP27 (**A**, **F**), and HSP90 (**A**, **G**). All values are expressed as mean ± standard deviation. Asterisks indicate significant differences (^ns^*P* > 0.05, *P < 0.05, and **P < 0.01) between the control (con) and treatment groups

We detected the expression of HSP27, HSP60, HSP70, HSP90, and HIF-1α in chicken jejunal tissue using western blotting. TS1 attenuated the inhibition of HSP70, HIF-1α, and HSP60 expression by SE in chicken intestinal tissue. The results in Fig. 5A-F indicate that SE treatment inhibited HSP60 (Fig. 5A, C), HSP70 (Fig. 5A, D) and HIF-1α (Fig. 5A, E) protein expression; however, TS1 significantly attenuated the SE-induced inhibition of HSP70 (*P* < 0.05), HIF-1α (*P* < 0.05), and HSP60 (*P* < 0.05) expression. Subsequently, we examined the mRNA expression of HSP70 (Fig. 5B) genes and found that TS1 significantly increased the expression of HSP70 decreased by SE. Thus, TS1 improved the expression of HSP70, HIF-1α, and HSP60 decreased by SE in chicken jejunal tissue. There was also no significant difference between the con group and TS1 group (*P* > 0.05). Results showed no significant changes in HSP27 (Fig. 5A, F) and HSP90 (Fig. 5A, G) expression.

### TS1 alleviated the abnormal expression of MAPK/PKC pathway-related proteins induced by *Salmonella* Enteritidis (SE)

**Fig. 6:**
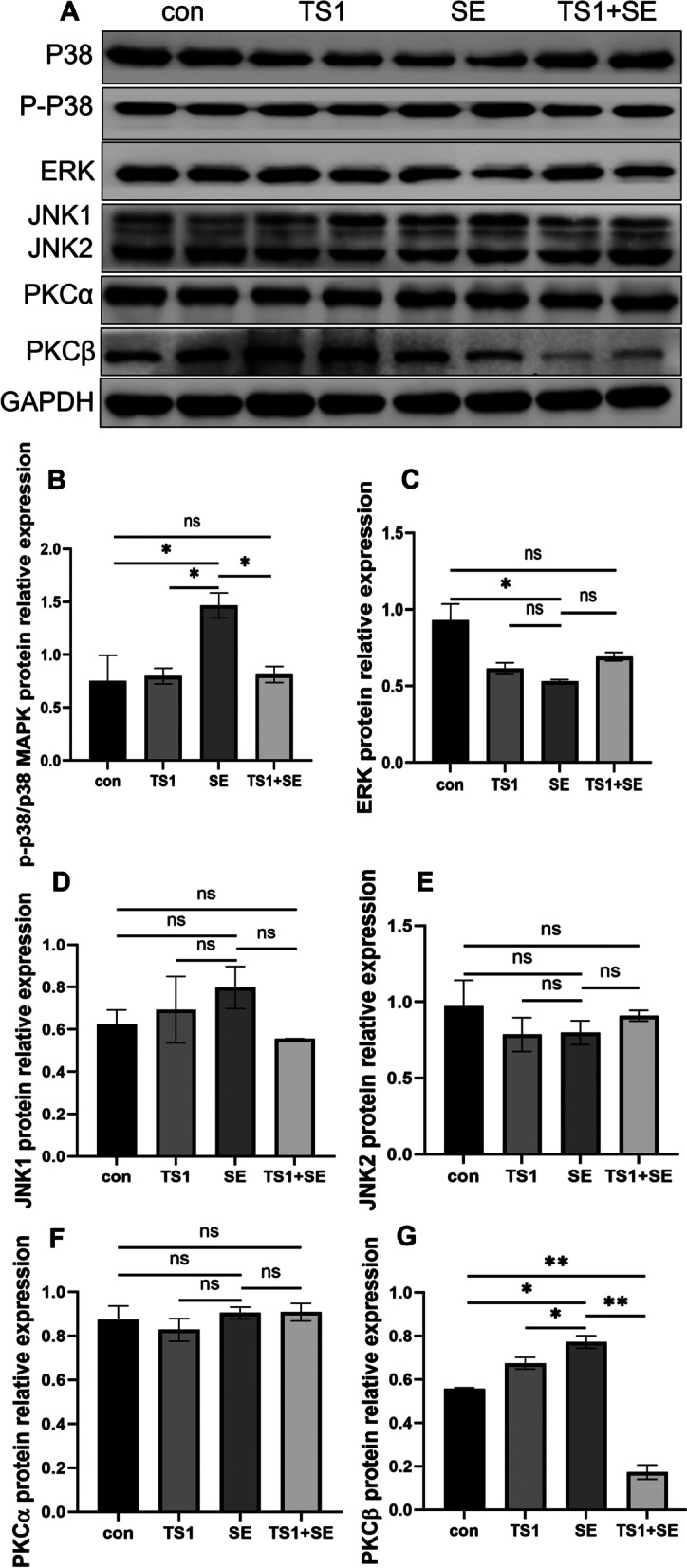
Effects of TS1 on MAPK/ERK pathway-related proteins—p38 MAPK, p-p38 MAPK (**A**, **B**), ERK (**A**, **C**), JNK (**A**, **D**, **E**), PKCα (**A**, **F**), and PKCβ (**A**, **G**). The levels of MAPK/ERK pathway-related proteins in different groups measured by western blotting. All values are expressed as mean ± standard deviation. Asterisks indicate significant differences (^ns^*P* > 0.05, *P < 0.05, and **P < 0.01) between the control (con) and treatment groups

We assessed the levels of ERK, JNK, and p38 MAPK and levels of phosphorylated p38 MAPK (Fig. 6). The SE group showed significantly higher expression levels of phosphorylated p38 MAPK than the con group (*P* < 0.05), whereas the TS1 + SE group showed significantly lower expression levels of phosphorylated p38 MAPK than the SE group (*P* < 0.05; Fig. 6A-B). The SE group showed significantly lower expression of ERK than the con group (*P* < 0.05), whereas the TS1 + SE group showed higher expression of ERK than the SE group (*P* > 0.05; Fig. 6A, D, E). For JNK levels, the differences between all groups were not significant (*P* > 0.05; Fig. 6A, D, E). No significant differences were observed between the con group and TS1 group (*P* > 0.05).

We also assessed the protein level of PKCα (Fig. 6A, F) and PKCβ (Fig. 6A, G). The effect of SE or TS1 on PKCα was not statistically significant (*P* > 0.05); however, the SE group showed significantly higher protein levels of PKCβ (*P* < 0.05) than the con group, whereas the TS1 + SE group alleviated this reduction. There were no significant differences observed between the con group and TS1 group (*P* > 0.05). These results indicate that TS1 increased the protein levels of p38 MAPK and phosphorylated p38 MAPK and decreased the protein level of PKCβ.

## Discussion

In the currently used intensive breeding system, broilers are exposed to heat, bacterial infection, and various other stressors that contribute to oxidative stress, especially in the intestine. ROS produced by oxidative stress damage nucleic acids, lipids, and proteins, alter the function of signaling proteins, and eventually lead to cell death [42]. Previous studies suggest that probiotics contribute to ROS removal, enzyme inhibition, metal chelation, and antioxidant enzyme synthesis [43]. Some studies have shown that probiotics improve the activity of SOD and GSH-Px and increase T-AOC while reducing the level of MDA [44]. In the present study, the activity of oxidative stress-related enzymes was also determined. Our results suggest that TS1 reduced the SE-induced increase in MDA content but enhanced the activity of SOD and GSH-Px and increased T-AOC. These findings are consistent with previous reports and indicate that TS1 protected poultry intestinal tissue from SE-induced damage by enhancing the function of the antioxidant system [45]. Oxidative stress causes over-inflammation, which leads to cell death or activates several apoptosis signaling pathways [46], which causes significant intestinal inflammatory cell infiltration, crypt loss, and epithelial cell destruction [47]. Liu et al. (2020) described the relationship between *Salmonella* infection and oxidative stress, which affects various key metabolic processes in the host, including DNA replication, regulation, and repair; substrate uptake; and RNA modification. Wang et al. reported that *Salmonella* infection in mice caused liver injury and inflammatory response; the mRNA levels of pro-inflammatory factors (IL-1β, IL-6, TNF-α, and IFN-γ) and chemokines (CCL2 and CCL3) in the liver increased significantly. In the present study, TS1 also reduced the SE-induced increase in the expression of pro-inflammatory cytokines (IL-1β, IL-6, TNF-α, IL-4, MCP-1) in the intestine and serum, which is consistent with previous findings and observations of lipopolysaccharide-induced enteritis in vitro [45, 48].

Hypoxia-inducible factor is a heterodimer comprising the regulatory subunit HIF-1α and the structural subunit HIF-1β with DNA binding activity. HIF-1α expression is induced by multiple pathways, and it is highly sensitive to oxygen concentration; therefore, it is considered the “master switch for hypoxic gene expression” [49]. HIF-1 inhibited ischemia–reperfusion-induced apoptosis in mouse cardiomyocytes under transient hypoxia. [50]. Frank et al. demonstrated that iNOS gene expression was induced by HIF-1 in hypoxic myocardium, and the amplification of the induction of iNOS gene and protein expression by IL-1 was also achieved through HIF-1 [51]. The present study found that the HIF-1 protein expression level in chicken intestines in the SE group was significantly lower than that in the con group, and HIF-1 expression level in the TS1 + SE group was significantly higher than that in the SE group. This finding suggests that TS1 mitigates the damage caused by oxidative stress by inducing the expression of HIF-1α. A study of pathological lesions showed that TS1 significantly alleviated intestinal tissue damage, including intestinal inflammatory cell infiltration, crypt loss, and epithelial cell damage.

Exposure to various stressors may cause a non-specific response, including at the protein level, especially changes in the expression of HSPs. HSP70 is one of the most widely studied HSPs. In the present study, the expression level of HSP70 was significantly lower in the SE group than in the con group and TS1 group and showed no significant difference between the con group and TS1 group. The HSP70 mRNA and protein levels decreased after SE treatment, indicating that HSP70 was depleted after being used as a molecular chaperone in the repair of misfolded proteins. Another plausible explanation for this finding is that *Salmonella enterica* stimulates the immune system, regulates the production of cytokines, and uses the host's chaperone proteins [52]. The TS1 + SE treatment increased the level of HSP70 in chicken intestinal tract, leading to less severe damage. These results also confirm previously reported findings that higher HSP70 levels have a protective effect in vivo and in vitro [33, 53]. Hasheimi et al. showed that heat stimulation elevated the expression of HSP70 in broiler chickens on day 42, whereas supplementation with *Zingiber officinale* and *Z. zerumbet* induced a higher expression of HSP70 to anti stress injury [54]. A previous study have shown that *Lactobacillus* ameliorates the suppression of HSP70 protein expression caused by *Salmonella enterica* in enterocyte-like Caco-2 cells [55]. Other HSPs were also studied in this study: HSP27 and HSP90 levels showed no significant differences among treatment groups. HSP27 and HSP90 act downstream of HSP70, whereas HSP60 improves folding and optimizes the maturation of key regulatory proteins [56]; this may explain why their levels did not change.

HSP70 and HSP60 function as chaperones for hundreds of proteins, including PKCβ. PKCβ is a predominant, conventional isoform of protein kinase C (PKC) and is a serine/threonine protein kinase involved in various cellular signal transduction pathways. PKC has been considered an important regulator of the pro-oxidant and pro-apoptotic function [57]. Disruption of the signaling pathways that activate and regulate PKCβ expression in response to cellular stress may therefore be an alternative approach to prevent oxidative damage [58, 59]. In the present study, the expression levels of PKCβ and phosphorylated p38 MAPK in the SE group were significantly higher than those in the con group, but the expression levels of PKCβ and phosphorylated p38 MAPK in the TS1 + SE group were significantly lower than those in the SE group. In animals, the p38 signaling pathway may be activated under several conditions, such as bacterial infection and high temperature, in turn activating cytokines such as growth factors and inflammatory factors. Moreover, p38 MAPK has been associated with PKC, which itself has been linked to oxidative stress and cellular damage [60]. The present study examined oxidation and inflammation-related proteins such as PKCβ, HIF1α, p38 MAPK, ERK, and JNK [42], and phosphorylated ERK and JNK proteins were also investigated; however, the primary antibodies were not suitable for chicken. The final results showed that after TS1 + SE treatment significantly decreased the levels of PKCβ and p38 MAPK proteins and increased the levels of HIF-1α and HSP70 proteins. These protein level changes were accompanied by less severe pathological damage in the intestinal tract, suggesting the protective effect of TS1 in broilers. In conclusion, our findings suggest that TS1 exerted its protective effect on broiler intestines by regulating MAPK/PKCβ, HIF-1α, and HSP70 expression, and its specific mechanism of action needs to be further explored.

## Conclusion

*Bacillus pumilus* TS1 alleviated oxidative stress damage caused by SE and attenuated the inflammatory response in broilers, and these effects were achieved through MAPK/PKC regulation of HSPs/HIF-1α.

## Materials and methods

### Animal ethics statement and treatment

The study protocol was approved by Nanjing Agricultural University Animal Care and Use Committee. AA308 white-feathered broilers were maintained in cages at the Laboratory Animal Center. The broiler feed base diet (New Hope Liuhe, 601) and drinking water were provided ad libitum. First, 120 one-day-old white-feathered broilers were randomly divided into four groups (con group, TS1 group, SE group, and TS1 + SE group) and pre-fed for 5 days. Each group contained three replicates, with 10 chickens per replicate. The feeding conditions of the four groups were identical; they were provided the same feed and water ad libitum. Broilers in the TS1 and TS1 + SE groups were fed with 1.4 × 10^7^ colony-forming units (CFU) per mL of TS1 by gavage for 15 days [61], whereas broilers in the con and SE groups were fed by gavage the same dose of PBS as the TS1 and TS1 + SE groups for 15 days. Then, the SE and TS1 + SE group chickens were intraperitoneally injected with 2.73 × 10^7^ CFU/mL SE (CICC, 21,513) for 3 days [34], whereas the con and TS1 group chickens was intraperitoneally injected with an equivalent dose of PBS for 3 consecutive days. Three days after SE and PBS injection, blood samples were collected from 10 randomly selected chickens in each group and stored in procoagulant tubes; then, the chickens were euthanized with pentobarbital, and the jejunum was immediately removed and divided into two parts. One part was placed in 4% paraformaldehyde to observe the ultrastructure, and the other part was flash-frozen in liquid nitrogen and stored at -80 C for further experimentation.

## *Isolation of* Bacillus pumilus

Fecal matter collected from healthy yaks from Danjun Horse Farm in Gansu Province was mixed with distilled water in a test tube and heated at 100 C in a water bath for 15 min; the mixture was streaked on a Luria–Bertani medium and incubated at 37 C for 24 h. In total, 49 isolates of intestinal bacteria were purified. The isolates were subjected to 16S genome sequencing; the resulting sequence were analyzed using BLAST in NCBI. The results showed that TS1 had 99% homology with *Bacillus pumilus*. Bacteriostatic experiments identified a strain with excellent acid and bile salt resistance [62], subsequently named TS1.

### Observation of jejunal morphological changes

Jejunal tissue samples were fixed in 4% paraformaldehyde (1:10 w/v) for at least 24 h. After sample dehydration and paraffin embedding, the tissues from each group were sectioned into 5 µm sections, dewaxed with xylene, and then stained with hematoxylin/eosin (Beyotime Institute of Biotechnology, Shanghai, China). Finally, the stained sections were observed using an Axio Imager A2 microscope (Zeiss, Germany) to examine the morphological differences among groups.

### Quantitative reverse transcription PCR of cytokines and HSP70

Total RNA was extracted from the jejunal tissue TRIzol (Vazyme R701-01, Nanjing, China) and reverse-transcribed to cDNA (Vazyme R223-01, Nanjing, China), according to the manufacturer’s instructions. RT-qPCR was performed using an AceQ qPCR SYBR Green Master Mix (Vazyme Q111-02, Nanjing, China) using a real-time PCR detection platform (Bio-Rad CFX96, USA). The primers for the target genes and reference gene (β-actin) used in this experiment are shown in Table 1. Only one peak for each PCR product was observed in the melting curve analysis. The results were normalized to the mean expression of β-actin, and the 2^−ΔΔCt^ method was used to calculate the relative mRNA expression [63].

### Enzyme-linked immunosorbent assay of serum cytokines

Blood was allowed to coagulate at 4  C overnight and then centrifuged at 4000 rpm for 10 min. The serum obtained was used to detect the levels of the following cytokines according to the manufacturer’s instructions of the respective detection kits: IL-1β (SEKCN-0153; Beijing Solarbio Science & Technology Co., Ltd., Beijing, China), IL-6 (SEKCN-0161; Beijing Solarbio Science & Technology Co., Ltd.), TNF-α (SEKCN-006; Beijing Solarbio Science & Technology Co., Ltd.), and monocyte chemoattractant protein (MCP)-1 (SEKCN-0169; Beijing Solarbio Science & Technology Co., Ltd.).

### Assessment of oxidative stress indexes

The serum samples obtained were also used to determine the levels of SOD (hydroxylamine method, A001-1), GSH-Px (colorimetric method, A005), and MDA (TBA method, A003-1–2) and T-AOC (colorimetric method, A015-1–2) using the respective detection kits according to the instructions of Nanjing Jiancheng Bioengineering Institute.

### Western blotting

The total protein of the jejunal tissue was extracted using RIPA lysis buffer with PMSF (Beyotime Institute of Biotechnology, Shanghai, China). Protein concentration of samples was determined using a bicinchoninic acid assay kit (Thermo Fisher, Shanghai, China) according to manufacturer’s instructions and adjusted to the same concentration across samples. The protein samples were added to 25 µL sodium dodecyl sulfate (SDS) loading buffer and denatured at 99 C for 10 min. Then, 5–6 µL of the above protein samples was separated on a 10% SDS–polyacrylamide gel by electrophoresis and then transferred to a polyvinylidene fluoride (PVDF) membrane in tris–glycine buffer containing 20% methanol at 4 C for 90 min. The membrane was blocked with 5% non-fat milk at 37 C for 2 h and incubated with anti-GAPDH (1:10,000, Abcam), anti-HSP27 (1:500, Abcam), anti-HSP60 (1:1000, CST), anti-HSP90 (1:1000, Enzo), anti-HSP70 (1:1000, Enzo), anti-HIF-1α (1:1000, Abbkine), anti-p38 MAPK (1:1000, CST), anti-phospho-p38 (1:2000, CST), anti-ERK (1:1000, CST), anti-JNK (1:1000, CST), anti-PKCα (1:10,000, Abcam), and anti-PKCβ (1:1000, Abcam) antibodies overnight at 4°C. Subsequently, the membranes were incubated with a horseradish peroxidase-conjugated secondary antibody against rabbit/mouse IgG (1:5000, ABclone). The protein bands were visualized using an enhanced chemiluminescence kit (Thermo Fisher, Shanghai, China). The protein gray values were then determined using ImageJ software, and the density of each band was normalized to that of its respective loading control (GAPDH). The obtained data were expressed as a ratio of the intensity of the protein in the experimental group to that of the corresponding protein in the con group.

### Statistical analysis

Statistical analysis of the data was performed using GraphPad Prism Version 9.0 software (GraphPad Software, Inc., La Jolla, CA, USA) using one-way analysis of variance with Tukey’s post-hoc test. All values are expressed as mean ± standard deviation. *P* < 0.05 was considered to indicate statistically significant differences between the con and treatment groups.

## Supplementary Information

**Additional file 1:** **Fig S1.** Figure 5 A original figure. **Fig S1.** Figure 6 A original figure.

## Data Availability

The datasets used and/or analyzed in the present study are available from the corresponding author on reasonable request.

## Abbreviations

SOD: Superoxide dismutaseand
GSH-PX: Glutathione peroxidase
MDA: Decreased and malondialdehyde
T-AOC: Total antioxidant capacity
IL-1β: Interleukin-1β
IL-6: Interleukin-6
TNFα: Tumor necrosis factorα
IL-4: Interleukin-4
MCP-1: Monocyte chemoattractant protein-1
HSR: Heat shock response
HSP: Heat shock proteins
SE: Salmonella enteritidis
PKCβ: Protein kinase Cβ
p38 MAPK: P38 mitogen-activated protein kinase
ERK: Extracellular regulated protein kinases
JNK: C-Jun N-terminal kinase
HIF-1α: Hypoxia inducible factor-1 alpha
