# Supplementary figures and images for: Bacillus pumilus TS1 alleviates Salmonella Enteritidis-induced intestinal injury in broilers
Source: BMC Vet Res. 2023 Feb 10;19:41. doi: 10.1186/s12917-023-03598-0 (PMC9912683; doi:10.1186/s12917-023-03598-0)

Fig S1 Figure 5 A original figures

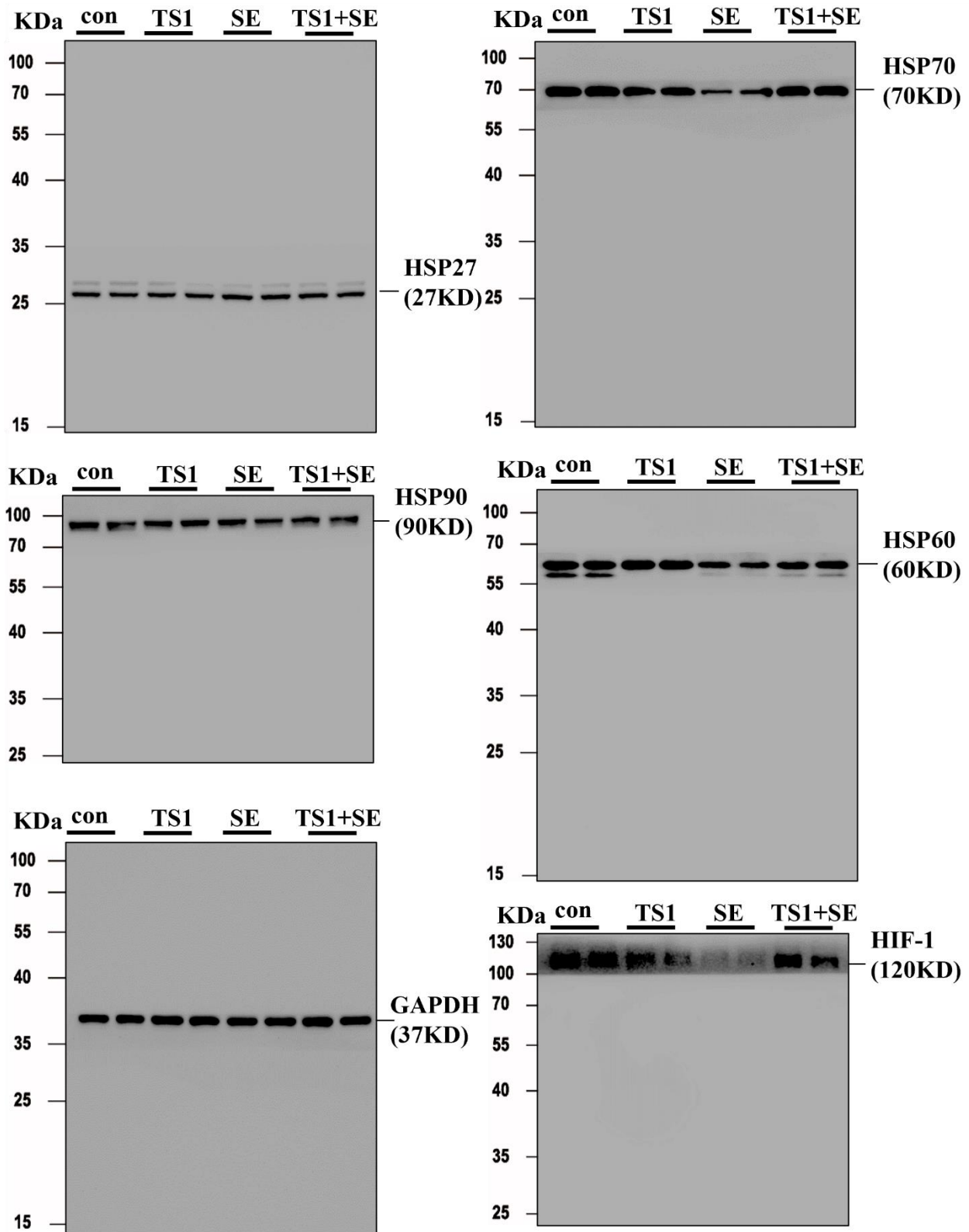

Fig S1 Figure 6 A original figures

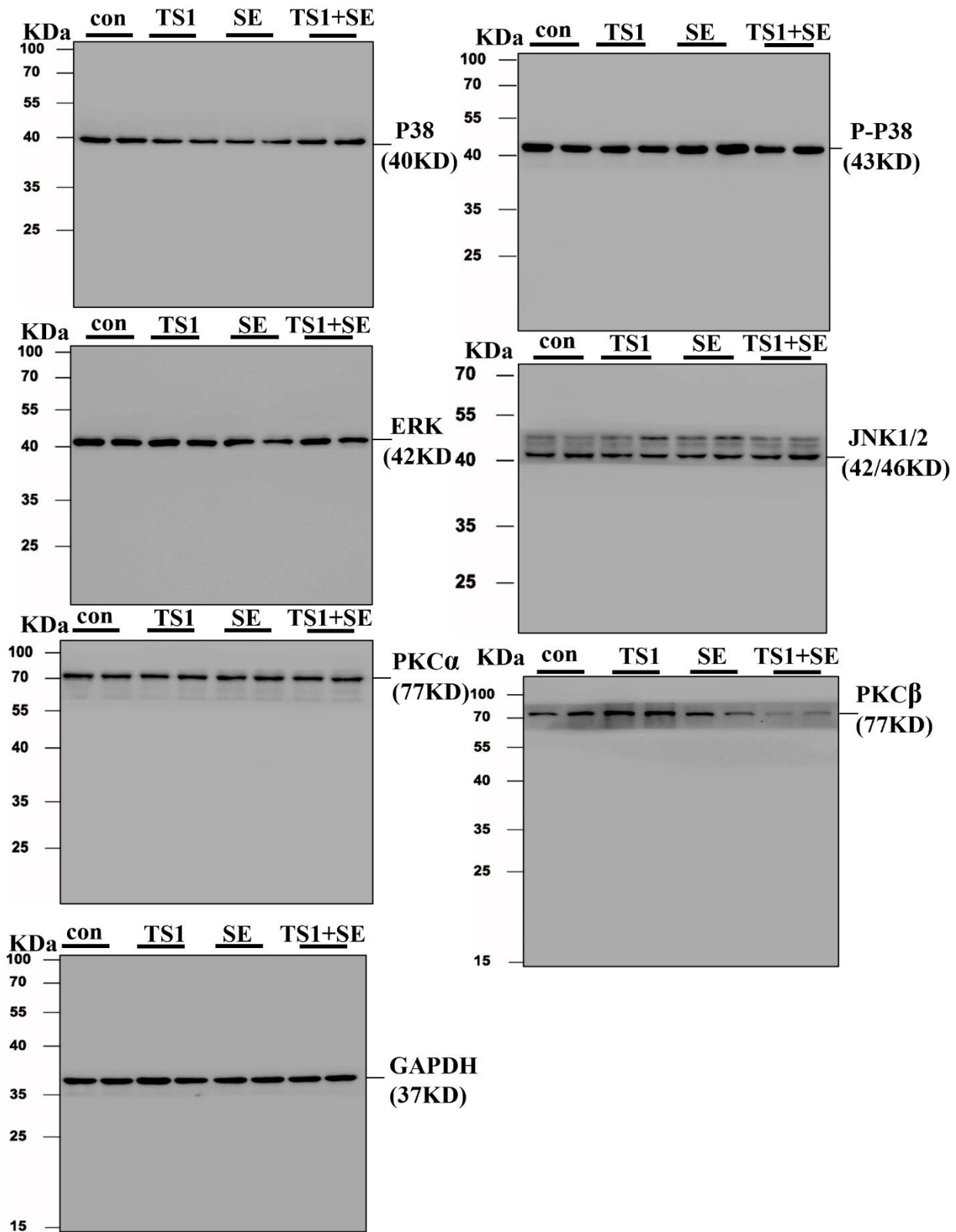

Supplement: Supplementary file 1 — Additional file 1: Fig S1. Figure 5 A original figure. Fig S1. Figure 6 A original figure. [file 12917_2023_3598_MOESM1_ESM.pdf]
